# Millimeter-Wave Permittivity Variations of an HR Silicon Substrate from the Photoconductive Effect

**DOI:** 10.3390/mi13101782

**Published:** 2022-10-19

**Authors:** Charlotte Tripon-Canseliet, Jean Chazelas

**Affiliations:** 1 LPEM, CNRS-PSL-Sorbonne Universités, 75005 Paris, France; 2 ULTIMETAS, 75020 Paris, France

**Keywords:** photoconductivity, microwave characterization, complex permittivity, free-space radar cross-section (RCS) measurements, semiconducting materials

## Abstract

The photoinduced microwave complex permittivity of a highly resistive single-crystal silicon wafer was extracted from a bistatic free-space characterization test bench operating in the 26.5–40 GHz frequency band under CW optical illumination at wavelengths of 806 and 971 nm. Significant variations in the real and imaginary parts of the substrate’s permittivity induced by direct photoconductivity are reported, with an optical power density dependence, in agreement with the theoretical predictions. These experimental results open the route to ultrafast system reconfiguration of microwave devices in integrated technology by an external EMI-protected and contactless control with unprecedented performance.

## 1. Introduction

The permittivity parameter plays a fundamental role in the propagation of electromagnetic waves within matter. Controlling this property remains an essential requirement in any communication system design. With the emergence of millimeter-wave technologies, for example, for beyond-5G communications, millimeter-wave radar systems, and sensors, accurate electromagnetic characterization of the material parameters becomes of fundamental significance [1], moreso when system reconfigurability is expected at the material level. Today, light interactions with matter [2] play a decisive role in controlling a system’s operation with a fast activation time [3]. There have been investigations of the optically induced complex dielectric constant change in the materials in the millimeter-wave range, as the K-band starts in SATCOM on-the-move and 5G-and-beyond communications. Among the tunability techniques, photoconductivity arises as one of the most potent phenomena with straightforward system integration. The microwave techniques that address the interaction of electromagnetic waves in the microwave range and the materials under test are nondestructive and instantaneous. Many approaches are available for the complex permittivity and conductivity characterization of the materials at high frequencies [4,5,6,7,8,9,10,11,12,13,14,15,16,17].

A noncontact method in microwave frequency has been developed to characterize a graphene sheet placed in the cross section of a rectangular waveguide. This latter requires the machining of materials to adapt them to measuring cells, and the presence of the gap between the sample and walls of the waveguide creates a capacitor parasite that can modify the extracted surface impedance. Resonant methods that use cavities have also been studied but are limited to single frequency measurements [18].

For decades, photoconductive effects have been exploited mainly for electrical signal-switching applications by a localized illumination implementation in integrated devices [19], with ON/OFF efficiency enhanced at very low dimensions, down to the nanoscale [20]. From a material point of view, the photosensitive part remains strongly dependent on the choice of semiconducting material and its photoconductive property requirements such as: (i) the bandgap energy windows (Eg = 0.8–1.5 eV) for light absorption exploitation from accessible commercial laser sources; (ii) high dark resistivity (ρ > 10${}^{7}$Ω·cm) for ON/OFF efficiency; (iii) a strong optical absorption coefficient; and (iv) the carrier dynamics tunability (lifetime τ–mobility μ) for time compatibility. Silicon (Si) and gallium arsenide (GaAs) materials are the most used material candidates because of their process management and costs. In this paper, we selected silicon as the most representative candidate for efficient demonstration in electronic systems, relying on its mature integrated technology [21], as well as for its broad optical absorption frequency band and coefficient inducing a micrometer-scale light penetration depth of this material, compared to other photoconductive materials [22].

## 2. Microwave Material Parameters Extraction from a Bistatic Experimental Setup

### 2.1. Permittivity Extraction Procedure

**Figure 1:**
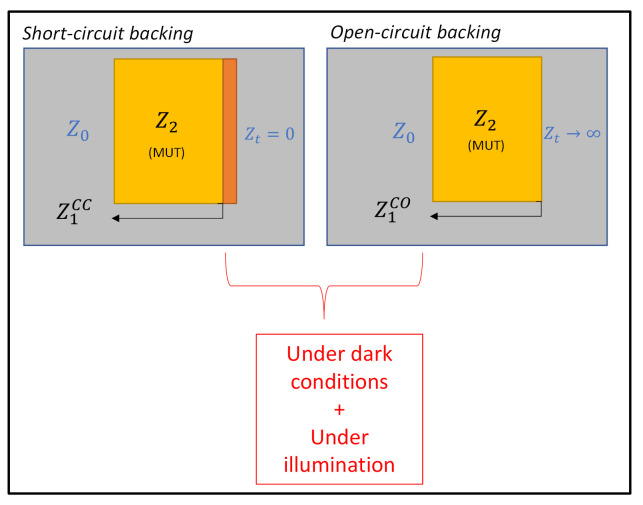
Calibration technique schematic for bistatic measurements in free space.

A specific free-space microwave bistatic characterization technique was developed for material parameters extraction, such as complex permittivity and permeability, under both microwave TM and TE mode excitations and at a specific incident angle θ. Using a calibration technique with a short- or open-circuit backing (layer 3) of an unknown Material Under Test (MUT) (layer 2) placed in a free-space environment (layers 1 and 4) with $Z_{0}$ equal to 377 Ω (Figure 1), direct identification of the MUT permittivity ($\epsilon_{2r}$) and permeability ($\mu_{2r}$) was achievable by de-embedding the calculations from the reflection coefficient measurement operating at the layer 1/layer 2 interface reference plane ($\Gamma^{ref_{p}lane}$). This procedure obtained direct access to the reference plane impedance values of the MUT under short-circuit $\left(Z_{1}^{CC}\right)$ or open-circuit $\left(Z_{1}^{CO}\right)$ backing (2) and, as a consequence, to the MUT impedance itself ($Z_{2}$) (1) and its associated permittivity value ($\epsilon_{2r}$), considering $k_{0}$ and $k_{z2}$ as the free-space and inside-the-MUT wave vectors’ magnitudes, respectively (2).

(1) $$Z_{2}^{2}=\frac{Z_{0}\cdot Z_{1}^{CC}\cdot Z_{1}^{CO}}{Z_{0}+Z_{1}^{CC}+Z_{1}^{CO}}$$

(2) $$\epsilon_{2r}=\frac{k_{z2}^{2}+k_{0}^{2}\cdot sin^{2}\theta }{k_{0}^{2}\cdot \mu_{2r}}$$

Developed analytically in [23], this technique can also be assisted by electromagnetic simulations that help in the suitable stacking of structures for de-embedding calculations, such as the MUT and reference loads’ dimensions, flatness requirements, and surface constraints.

### 2.2. Experimental Setup

**Figure 2:**
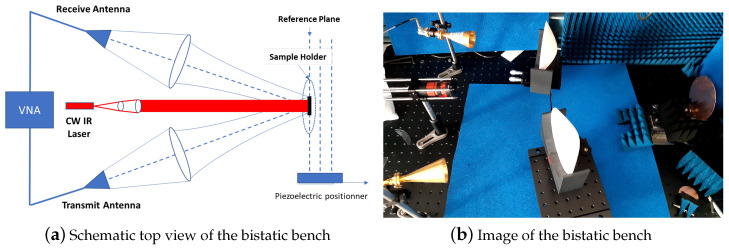
Schematic view (**a**) and image (**b**) of the dedicated free-space microwave bistatic characterization technique developed at PSL/ESPCI.

Microwave measurements were performed from the free-space bistatic experimental setup (Figure 2), in which a laser source was used. A wideband Vectorial Network Analyzer (VNA) was connected to WR28-type Tx/RX horn antennas, which propagated and collected the microwave signal coming to and from the MUT sample after microwave lens focusing. The MUT sample was placed at a far-field distance from the horn antennas with θ equal to 10° and mounted in a piezoelectric fixture demonstrating a 10 nm displacement accuracy in all spatial directions. As a first step, an SOLT calibration procedure was operated at the antennas’ input reference plane in coaxial termination, providing a measurement accuracy of 0.01 dB and 2deg in magnitude and phase on the $S_{21}$ parameter standing for the reflection coefficient measurement in this configuration. As a second step, in order to compensate for the propagation losses and delay from the antennas to the MUT reference plane, the reflection coefficient extraction at the reference plane ($\Gamma^{refplane}$) was performed twice, first replacing the MUT sample with a metallic place with same diameter ($S_{21}^{Metal}$) and, second, without any sample ($S_{21}^{Antennas}$), leading to (3).

In order to match the squared or spherical centimeter-scale MUT surface characterization with the mandatory far-field conditions, a 26.5–40 GHz operational frequency band was selected [11,24,25].

(3) $$\Gamma^{refplane}=\frac{S_{21}^{Total}-S_{21}^{Antennas}}{S_{21}^{Antennas}-S_{21}^{Metal}}$$

## 3. Microwave Permittivity, Conductivity, and Photoconductivity Definitions in Semiconducting Materials

The photoconductive effect is based on the phenomenon of photon absorption into a semiconductor material [20]. Under optical illumination carrying a photon energy superior to the energy bandgap Eg of the material, free carriers, either electrons or holes, are photogenerated in the illuminated area, changing the material conductivity locally by a value of Δσ [26,27,28,29]. Furthermore, the general distribution in space of the material photoconductivity results from the superposition of the absorption parameter (α) and the ambipolar carrier diffusion length (L) (4), according to Platte and B. Sauerer [30]. A global approximation, which also implements the surface recombination velocity of the photogenerated carriers $v_{s}$ and their recombination lifetime $\tau_{r}$ can be applied.

(4) $$L=\sqrt{\frac{2k_{B}\cdot T\tau_{r}}{q}\frac{\mu_{n}\cdot \mu_{p}}{\mu_{n}+\mu_{p}}}$$

(5) $$\Delta \sigma_{m}=\frac{\Delta \sigma_{opt}}{1+\alpha L}{\left(\frac{1}{\alpha L}\frac{\alpha L^{2}+v_{s}\tau_{r}}{L+v_{s}\tau_{r}}\right)}^{\frac{-\alpha L}{1-\alpha L}}$$

(6) $$\Delta \sigma_{opt}=KS\alpha P_{opt}\tau_{r}$$

(7) $$K=q\frac{\lambda }{hc}\left(\mu_{n}+\mu_{p}\right)\left(1-R\right)$$

Considering (5)–(7), where *q* is the electronic charge, *h* is the Planck’s constant, *c* is the velocity of light in a vacuum, $\mu_{n}$ and $\mu_{p}$ are the mobilities of electrons and holes, respectively, *R* is the surface reflectivity, and *S* is the relative spectral response of the semiconductor material exhibiting a peak response at λ, a maximum photoconductivity value $\Delta \sigma_{m}$ is then achievable at an effective penetration depth.

### 3.1. Analytic Modeling of Permittivity

Assuming a classical dielectric function definition, such as the Drude model [31], the frequency-dependent permittivity expression applied to conductive materials (8) can be written as a generic complex formula that takes into account the material conductivity variation σ(ω) with angular frequency ω (9)

(8) $$\epsilon \left(\omega \right)=\epsilon_{s}-i\frac{\sigma (\omega )}{\omega \epsilon_{0}}$$

(9) $$\sigma \left(\omega \right)=\frac{\sigma_{0}}{1-i\omega \tau }$$

(10) $$\sigma_{0}=\tau \cdot q^{2}\left(\frac{n}{m_{e}^{*}}+\frac{p}{m_{h}^{*}}\right)$$

(11) $$\varepsilon \left(\omega \right)=\varepsilon^{\prime }\left(\omega \right)+i\varepsilon^{\prime \prime }\left(\omega \right)$$

(12) $$\epsilon^{\prime }\left(\omega \right)=\epsilon_{s}+\frac{\sigma_{0}\tau }{\epsilon_{0}\left(1+\omega^{2}\tau^{2}\right)}$$

(13) $$\epsilon^{\prime \prime }\left(\omega \right)=-\frac{\sigma_{0}}{\omega \epsilon_{0}\left(1+\omega^{2}\tau^{2}\right)}$$

where $\epsilon_{S}$ represents the permittivity in a static regime, τ represents the collision time, $\sigma_{0}$ represents the dc material conductivity, *n* and *p* are the concentrations of the conduction electrons and valence holes, respectively, and m* is the electron effective mass (11). In most cases, the ωτ product is neglected, compared to 1, leading to a σ(ω) to $\sigma_{0}$ approximation. Here, in the microwave regime, it is essential to notice that the σ(ω) definition cannot be reduced to a real contribution $\sigma_{0}$ and must be fully described by (9). In consequence, a consistent imaginary part of σ(ω) must be considered [32] leading to the complex expression of ϵ(ω) (11) in real (12) and imaginary (13) parts.

### 3.2. Analytic Modeling of the Permittivity, including Photoconductive Parameters

Under absorptive illumination, $\sigma_{0}$ was then incremented to $\sigma_{0}+\Delta \sigma_{m}$. Accordingly, this light/matter interaction also induced a change in permittivity, as defined in (12), (13) for both real and imaginary parts, as suggested in [27], with the implementation of the Platte model by introducing (5).

## 4. Experimental Microwave Permittivity Extraction of the HR Silicon Substrate

The experimental complex permittivity data of a highly resistive (HR) silicon wafer were extracted under various optical power densities and wavelengths (Figure 2). Variable beam diameter collimated illumination was applied on the top face of the MUT by a LUMICS Mini-Ocean laser source, which delivers a high-power CW optical signal at 806 and 971 nm optical wavelengths. The MUT was a two-inch diameter commercial HR Si substrate providing a dark resistivity $\rho_{0}$ of $10^{5}$ Ω·cm or a dark conductivity $\sigma_{0}$ equal to $10^{-3}$ S/m and a thickness of 280 µm.

### 4.1. Under Dark Conditions

First, to validate the two-step calibration procedure, the initial set of data was collected from the HR Si substrate placed as the MUT under dark conditions. From the measurements, we identified the experimental values for the τ of 1 ps and for $\epsilon_{\infty }$ of 10.2 from the $\sigma_{0}$ (11) and $\epsilon^{\prime }\left(\omega \right)$ (12) data, respectively. At this step, it must be noticed that a conductivity offset value $\sigma_{offset}$ of $\sigma_{0}$, evaluated to 2.2 S/m, must be considered from the global experimental environment.

**Figure 3:**
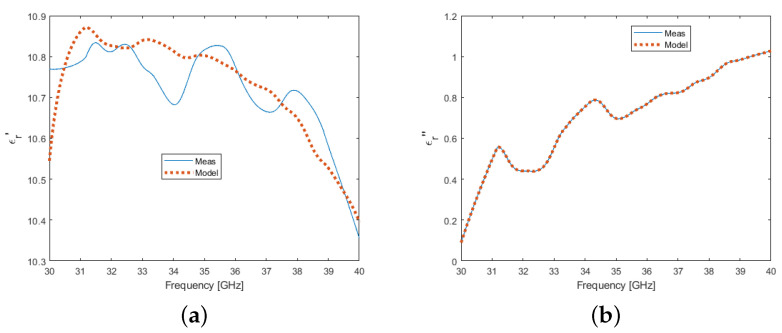
Microwave silicon permittivity under dark conditions—Experimental and simulation data in real (**a**) and imaginary (**b**) parts.

Assuming these statements, the frequency-dependent experimental complex permittivity data demonstrated a very good agreement with the theoretical Drude model predictions as depicted in (Figure 3) for both real and imaginary parts, which validated the experimental setup and the de-embedding calculations’ operability.

### 4.2. Under Optical Illumination

According to previous theoretical approaches [33], the closed-form expression of the frequency-dependent complex permittivity is defined by (15), (16) in which the photoconductive effect is identified as an additional parameter in the MUT conductivity $\Delta \sigma_{m}$ compared to the off-state conductivity $\sigma_{o}$ (14).

(14) $$\sigma_{on}=\sigma_{0}+\Delta \sigma_{m}$$

with

(15) $$\epsilon_{on}^{\prime }\left(\omega \right)=\epsilon_{\infty }+\frac{\sigma_{on}\tau }{\epsilon_{0}\left(1+\omega^{2}\tau^{2}\right)}$$

(16) $$\epsilon_{on}^{\prime \prime }\left(\omega \right)=-\frac{\sigma_{on}}{\omega \epsilon_{0}\left(1+\omega^{2}\tau^{2}\right)}$$

Taking into account the $\sigma_{offset}$ value, (15), (16) can be modified by (17), (18).

(17) $$\epsilon_{on}^{\prime }\left(\omega \right)=\epsilon_{\infty }+\frac{(\sigma_{offset}+\sigma_{on})\tau }{\epsilon_{0}\left(1+\omega^{2}\tau^{2}\right)}$$

(18) $$\epsilon_{on}^{\prime \prime }\left(\omega \right)=\frac{\sigma_{offset}-\sigma_{on}}{\omega \epsilon_{0}\left(1+\omega^{2}\tau^{2}\right)}$$

**Figure 4:**
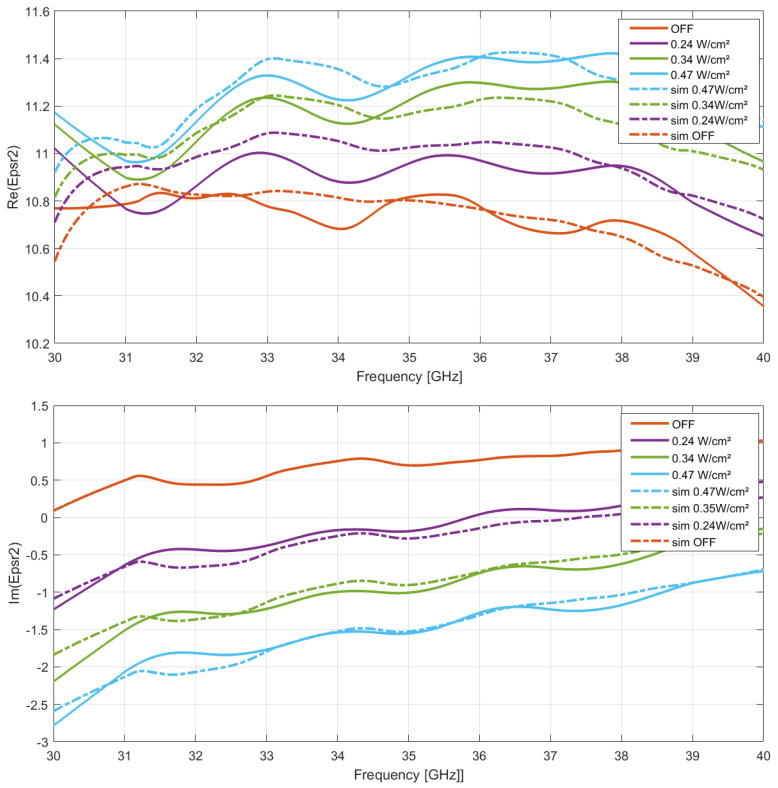
Microwave silicon permittivity variations. Experimental (solid) and simulation (dashed) data comparison under optical illumination in real and imaginary parts: $\lambda_{opt}$ = 806 nm.

**Figure 5:**
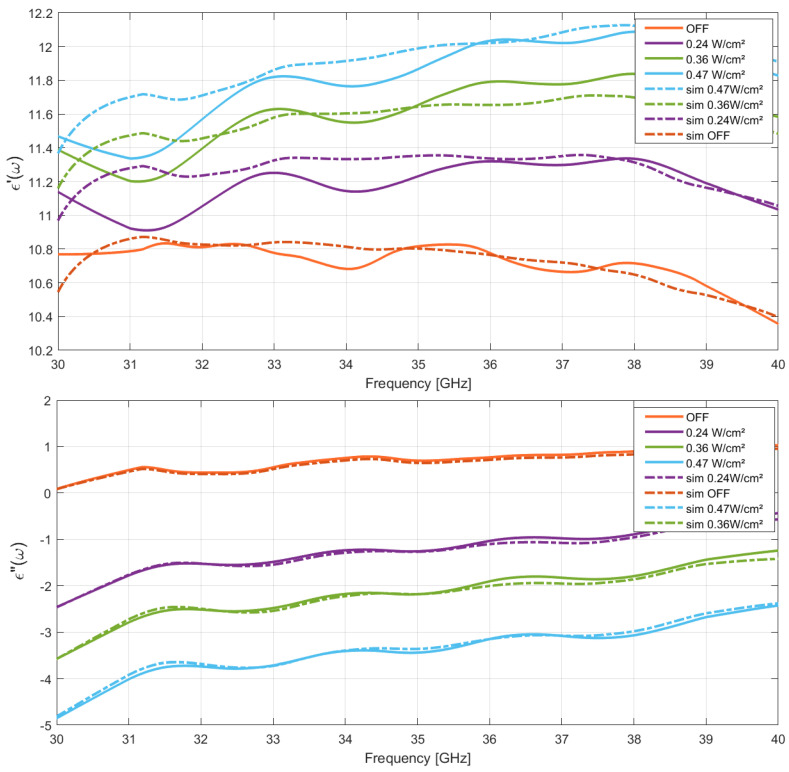
Microwave silicon permittivity variations. Experimental (solid) and simulation (dashed) comparison under optical illumination in real and imaginary parts: $\lambda_{opt}$ = 971 nm.

The experimental results of the real and imaginary parts variations in the microwave frequency of the HR Si permittivity are presented in Figure 4 and in Figure 5 under various optical power densities (OPD) and 806/971 nm optical wavelengths. As expected, from the classical material conductivity model, a linear variation in $\epsilon^{\prime \prime }$ with OPD dependency was observed as a comprehensive change related to the photoconductive effect at each optical wavelength. Furthermore, a significant nonlinear variation in the real part of the permittivity with OPD dependency was simultaneously detected at the highest microwave frequency, attributed to the nonnegligible ωτ product impact.

### 4.3. Physical Interpretation from Drude Model

In order to extract complementary material parameters, feedback analytic simulations using the Platte theoretical model from (14) and (15) were conducted from the experimental permittivity data under low OPD illumination. From the calculation of $\Delta \sigma_{m}$, the extracted values of the excess carrier lifetime ($\tau_{r}$), carrier diffusion length (L), surface recombination velocity ($v_{s}$), spectral response S, and absorption coefficient α under varying optical power densities (OPD) are summarized in Table 1 for each optical wavelength. The extracted values allowed agreement of the measurement and calculated data from the proposed modified Drude model to be extended to the photoconductive materials.

As an example, the detailed complex permittivity variations values of HR silicon under light illumination at 971 nm and 806 nm are summarized in Table 2 and Table 3 for a microwave frequency of 40 GHz. As observed from the simulations, unprecedented significant changes in the real part of the HR silicon permittivity value under different OPDs were revealed, because of the τ value contribution with respect to the microwave frequency. The highest performances were also obtained at larger optical wavelengths as expected from the Platte model, an effective maximum permittivity tuning from 7 to 13.8% in the permittivity real part was measured under an OPD of 0.47 W/cm^2^ at 806 or 971 nm optical wavelengths, respectively. Concerning the imaginary part of the permittivity, as expected from the classical theory on photoconductive effect, an enhancement of 1.75 and 3.45 points was observed under identical optical parameters as related to an effective $\Delta \sigma_{m}$ contribution in this type of material.

## 5. Experimental Microwave Photoconductivity Extraction under Optical Illumination

**Figure 6:**
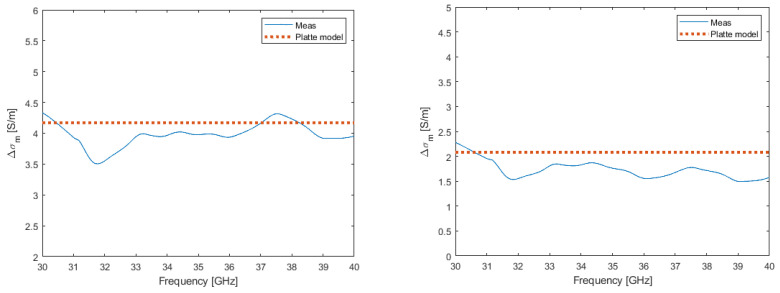
Extracted photoconductivity parameter variation with microwave frequency under optical illumination of OPD = 0.24 W/cm^2^ and optical wavelengths of 971 nm (**left**) and 806 nm (**right**).

From these measurements, the substrate conductivity’s relative change $\Delta \sigma_{m}$ by the photoconductivity, from its original dark conductivity $\sigma_{0}$ evaluated at 2.2 S/m under different OPD states, was extracted from the experimental data of $\epsilon_{on}^{\prime \prime }\left(\omega \right)$ in the microwave frequency (14). The evolution in the frequency of the photoconductivity under CW optical illumination followed a quasi-constant regime, as stated by the Platte model in (5); see, for example, in Figure 6, which corresponded to an OPD of 0.24 W/cm^2^. The detailed data values reported in Table 4 and Table 5, at two optical wavelength excitations and under different OPDs, for a microwave frequency of 40 GHz, exhibited a grood agreement with the theoretical values.

## 6. Discussion and Conclusions

Using a free-space bistatic experimental setup and based on de-embedding approaches, we measured the complex permittivity of the high-resistivity silicon substrate in the millimeter-wave range, from 30 to 40 GHz. We extended these experiments to include an optical illumination of the substrate in order to evaluate the variation in the permittivity of Si under optical illumination. An increase in the real part of the permittivity was experimentally demonstrated, and a modified Drude model based on the induced photoconductivity was developed. It fit the experimental results at two different wavelengths, 971 nm and 806 nm. The results open new opportunities for the development of optoelectronically controlled millimeter-wave devices and systems.

## Figures and Tables

**Table 1:** Theoretical optical parameters used for the Platte model.

$\lambda_{\mathrm{opt}}$	$\tau_{r}$	*L*	$v_{s}$	α	*K*	*S*
(nm)	(μs)	(μm)	(cm/s)	m${}^{-1}$	(m${}^{2}$/V${}^{2}$)	(a.u)
971	5.8	116	400	$10^{4}$	0.1	0.7
806	0.8	39	100	$10^{5}$	0.09	0.8

**Table 2:** Experimental permittivity shift values obtained at f = 40 GHz and optical illumination at $\lambda_{opt}$ = 971 nm.

OPD	$\Delta \epsilon_{r}^{\prime }$	$\Delta \epsilon_{r}^{\prime \prime }$
(W/cm^2^)	(a.u)	(a.u)
0.24	0.68	1.46
0.36	1.23	2.27
0.47	1.47	3.45

**Table 3:** Experimental permittivity shift values obtained at f = 40 GHz and optical illumination at $\lambda_{opt}$ = 806 nm.

OPD	$\Delta \epsilon_{r}^{\prime }$	$\Delta \epsilon_{r}^{\prime \prime }$
(W/cm^2^)	(a.u)	(a.u)
0.24	0.29	0.55
0.36	0.61	1.18
0.47	0.75	1.75

**Table 4:** Experimental photoconductivity values obtained at f = 40 GHz and $\lambda_{opt}$ = 971 nm.

OPD	$\Delta \sigma_{m}\left(meas\right)$	$\Delta \sigma_{m}\left(theor\right)$
(W/cm^2^)	(S/m)	(S/m)
0.24	3.95	4.17
0.36	6.5	6.29
0.47	9.07	8.17

**Table 5:** Experimental photoconductivity values obtained at f = 40 GHz and $\lambda_{opt}$ = 806 nm.

OPD	$\Delta \sigma_{m}\left(meas\right)$	$\Delta \sigma_{m}\left(theor\right)$
(W/cm^2^)	(S/m)	(S/m)
0.24	1.57	2.08
0.36	3.33	3.41
0.47	4.61	4.73

## Data Availability

Not applicable.
